# Psychological predictors of sports injuries in elite ski athletes: a multidimensional analysis of personality, anxiety, depression and inflexibility

**DOI:** 10.3389/fpsyg.2025.1698313

**Published:** 2025-11-03

**Authors:** Sara Mogedano-Cruz, Vicente Javier Clemente-Suárez, Rafael Jácome-López, Fernando García-Sanz, Laura González-Fernández, Ángel González-de-la-Flor, Carlos Romero-Morales

**Affiliations:** Universidad Europea de Madrid, Faculty of Medicine, Health and Sports, Department of Physiotherapy, Villaviciosa de Odón, Madrid, Spain

**Keywords:** alpine skiing, sports injuries, neuroticism, mental health, psychological profile, injury prevention

## Abstract

**Introduction:**

Injuries among elite alpine sky athletes can lead to both physical and psychological consequences. While previous studies have pointed out how emotional factors can influence the risk of injuries, there has not been much investigation into how different psychological elements relate to the overall injury burden.

**Methods:**

This study aimed to evaluate how traits like neuroticism, anxiety, depression, psychological inflexibility, loneliness, and conscientiousness predict the frequency and severity of injuries in professional skiers. Additionally, it sought to identify psychological profiles that might indicate a higher risk of injury. We conducted a cross-sectional, observational, and analytical study involving 50 active professional alpine skiers. The data were analyzed using Poisson regression for injury frequency, multiple linear regression for perceived injury severity, and K-means cluster analysis to identify different psychological profiles.

**Results:**

The findings revealed that neuroticism [Exp (β) = 1.15, *p* = 0.026] and psychological inflexibility [Exp (β) = 1.09, *p* = 0.041] were significant predictors of injury frequency. Perceived injury severity was associated with depressive symptoms (β = 0.34, *p* = 0.009), inflexibility (β = 0.26, *p* = 0.034) and neuroticism (β = 0.21, *p* = 0.048). We identified three distinct psychological profiles: resilient, average and vulnerable, with the vulnerable group experiencing the highest injury burden (*p* < 0.05).

**Conclusion:**

Certain psychological traits, especially neuroticism, inflexibility, and depressive symptoms are associated with an increased risk and severity of injuries. By identifying these psychological risk profiles, we can develop targeted interventions to prevent injuries and support recovery. It’s essential to incorporate psychological assessments into health and performance programs for athletes engaged in high-demand sports.

## Introduction

Snow sports such as alpine skiing are becoming more common and available around the world. It is estimated that there are over 200 million active skiers globally (Davey et al., 2019). However, these disciplines have a risk of injury, particularly in high-level competitive contexts (Patrick et al., 2015; Schneider, 2003; Stenroos and Handolin, 2015). In alpine skiing, the most common injuries tend to affect the knee, shoulder, and wrist, with an incidence rate of about two to five injuries occurring for every 1,000 ski days (Davey et al., 2019; Burtscher et al., 2008; Coury et al., 2013). These injuries can have a significant physical impact, but they also carry psychological consequences that can impact well-being, motivation and the athletic careers of elite skiers (Finkenzeller et al., 2022; Gao et al., 2024).

Traditionally, the focus on injury risk in winter sports has been on biomechanical, environmental, and technical aspects (Seifert et al., 2009; Koller et al., 2015). However, in recent years, there has been a growing interest in how psychological factors might predict injury susceptibility (Nicolò et al., 2017). Beyond isolated findings, several theoretical models have attempted to clarify how physiological and situational variables interact with psychological factors to influence injury risk. According to the Wiese-Bjornstal et al. (1998) stress-injury model, psychological responses to stress—shaped by coping mechanisms, personal traits, and the athlete’s history—can directly impact both the risk of sustaining an injury and the subsequent recovery process. Similarly, the biopsychosocial model proposed by Olmedilla Zafra and Garcia-Mas (2012) highlights the dynamic interaction between emotional, contextual, and cognitive factors in injury predisposition, showing how psychological vulnerability can influence physical risk (Olmedilla et al., 2018). By combining these theoretical frameworks, we can better understand how attributes such as neuroticism or psychological rigidity may contribute to a higher likelihood of injuries (Andersen and Williams, 1988). These traits can shape how athletes perceive risk, maintain focus, regulate their emotions and manage themselves, especially in high-speed and technically challenging sports like alpine skiing (Birrer and Morgan, 2010; Eather et al., 2023).

In particular, athletes with high levels of neuroticism tend to experience more negative emotions, lower stress tolerance and greater emotional reactivity, which can lead to mistakes that result in injuries (Xiao, 2024). Similarly, elevated anxiety has been linked to issues with attention and decision-making when under pressure (Korkutata et al., 2024). Psychological inflexibility reflects difficulty in managing unpleasant internal experiences, which can result in maladaptive responses to pain, fatigue or adversity (Junge, 2000; Arbinaga Ibarzábal, 2025). This is particularly important in high-level alpine skiing, where extreme physical conditions, social isolation, and intense mental pressures come together, making it crucial for developing effective prevention strategies (Gao et al., 2024).

Although previous studies have examined the impact of certain psychological factors separately, an important gap remains in understanding how these variables combine and cluster into psychological risk profiles that could affect both the severity and frequency of injuries. Most existing research has not incorporated these aspects into a comprehensive theoretical framework, which limits their explanatory power and practical applicability. Our analysis adapts a multidimensional perspective grounded in well-established theoretical models, such as the biopsychosocial model proposed by Olmedilla et al. (2018) and the stress-injury model developed by Wiese-Bjornstal et al. (1998).

This study aims to address this gap by analyzing a broad set of psychological variables (neuroticism, anxiety, depression, psychological inflexibility, loneliness, and conscientiousness) as predictors of injury frequency and severity in professional skiers. Rather than just examining each factor on its own, the research also seeks to identify distinct psychological profiles through cluster analysis, which can help highlight subgroups of athletes who may be more psychologically vulnerable. We hypothesize that athletes with higher levels of neuroticism, emotional symptoms and psychological inflexibility, along with lower levels of conscientiousness, will face a greater injury burden. Additionally, we expect that skiers who fit into a “vulnerable” psychological profile will report significantly higher rates of injury frequency and severity compared to those with more resilient profiles.

## Methods

### Study design

A cross-sectional, observational, and analytical study was conducted in accordance with the guidelines of the Strengthening the Reporting of Observational Studies in Epidemiology (STROBE) checklist (Cuschieri, 2019). The aim was to explore the relationship between psychological variables and injury burden in elite alpine skiing athletes. Data collection was carried out remotely through a standardized online survey administered over a two-month period.

### Participants

Participants were recruited from national and regional alpine skiing teams through direct contact with coaches and team staff. A total of 50 professional athletes (34 males, 16 females) participated in the study. All were actively competing in elite-level sports, primarily alpine skiing, at the time of participation. The mean age of the sample was 27.6 years (SD = 4.3), with a mean height of 176.3 cm (SD = 8.9), mean weight of 72.6 kg (SD = 10.3) and an average body mass index (BMI) of 23.4 (SD = 2.3). Participants reported an average of 9.1 years (SD = 3.2) of competitive experience in their sport.

Inclusion criteria required athletes to be over 18 years old, currently engaged in national or international competition, and to have sustained at least one injury in the past year. Exclusion criteria included the presence of any diagnosed psychiatric or neurological conditions that could compromise the accuracy of self-report data. No athletes were excluded after recruitment, and no dropouts occurred during data collection. All participants provided informed consent prior to participation, and the study protocol received approval from the Institutional Ethics Committee, following the ethical standards of the Declaration of Helsinki.

### Sample size calculation

The sample size was estimated using G*Power 3.1 software, based on a multiple regression model with six predictors, an alpha level of 0.05, a statistical power of 80% (1–β = 0.80) and a medium effect size (*f*^2^ = 0.15). The analysis indicated a minimum sample size of 48 participants; thus, the final sample of 50 was considered adequate to ensure sufficient statistical power for the main analyses.

### Variables

Variables were divided into two broad categories: psychological variables and injury-related variables. The selection of instruments was based on relevant previous studies (Martín-Rodríguez et al., 2021; Rodriguez-Besteiro et al., 2023).

Psychological variables included personality traits, anxiety, psychological inflexibility, perceived loneliness, and depressive symptoms.

Personality traits were assessed using the Spanish version of the Big Five Inventory (BFI-44), developed by Benet-Martínez and John (1998), which evaluates five major dimensions: extraversion, agreeableness, conscientiousness, neuroticism and openness to experience. The Spanish adaptation has demonstrated robust psychometric properties, with internal consistency coefficients ranging from *α* = 0.77 to 0.83 across dimensions in Spanish populations.

Anxiety levels were measured with the 10-item short version of the State–Trait Anxiety Inventory (STAI), developed by Marteau and Bekker (1992), with the Spanish validation showing excellent reliability (*α* = 0.82) for state anxiety (Guillén-Riquelme and Buela-Casal, 2011). This abbreviated version has shown good reliability and validity for efficiently capturing transient anxiety symptoms.

Psychological inflexibility was assessed with the Acceptance and Action Questionnaire-II (AAQ-II), developed by Bond et al. (2011). It is a 7-item instrument designed to measure experiential avoidance and unwillingness to remain in contact with aversive thoughts and emotions. Higher scores indicate greater inflexibility. The Spanish version of the AAQ-II demonstrated strong internal consistency (*α* = 0.88) and good construct validity (Ruiz et al., 2013). This instrument is widely used in both clinical and non-clinical samples.

Perceived loneliness was measured with the UCLA Loneliness Scale (Version 3) (Russell, 1996). The Spanish adaptation used in this study shows adequate reliability (*α* = 0.89) (Fernández, 1993). This tool assesses subjective feelings of social isolation and disconnection and is commonly used in sports and mental health research (Russell, 1996).

Finally, depressive symptoms were measured using the Zung Self-Rating Depression Scale (Zung, 1965), a 20-item self-report inventory that evaluates affective, psychological, and somatic symptoms associated with depression. This scale has been validated in Spanish-speaking populations, which showed a Cronbach’s alpha of *α* = 0.84 (Zung, 1965; Biggs et al., 1978).

For injury-related variables, participants were asked to report the number of injuries sustained during the past competitive season, the anatomical location and type of their most serious injury, the time required for recovery (in months), and to rate the perceived severity of the injury on a Likert-type scale from 0 (not severe) to 10 (extremely severe).

### Statistical analysis

All statistical analyses were conducted using IBM SPSS Statistics (version 27) and R (version 4.3.1). Prior to analysis, data were screened for missing values, outliers, and assumptions of normality. Descriptive statistics were computed for all variables. Pearson’s correlations were used to examine bivariate associations between psychological traits and injury-related outcomes.

Injury frequency, treated as a count variable, was analyzed using Poisson regression with psychological predictors (neuroticism, conscientiousness, anxiety, psychological inflexibility, depression, and loneliness) and demographic covariates (age and sex). Injury severity, measured on a continuous scale, was analyzed via multiple linear regression using the same predictors.

A K-means cluster analysis was conducted to identify distinct psychological profiles based on scores from the Big Five Inventory, STAI, AAQ-II, UCLA, and Zung scales. The optimal number of clusters was determined by visual inspection of the elbow plot and silhouette values. Group differences in injury outcomes across psychological profiles were tested using one-way ANOVAs and *post hoc* comparisons with Bonferroni correction.

Multicollinearity among predictors was assessed using variance inflation factors (VIFs), with values below 2.0 considered acceptable. Statistical significance was set at *p* < 0.05 for all tests. Effect sizes [e.g., β, Exp (β), η^2^] were reported to facilitate interpretation of results.

## Results

### Descriptive and correlational analysis

Descriptive statistics were computed for all psychological and injury-related variables. The sample showed moderate mean levels of neuroticism (*M* = 3.9 ± 1.0), anxiety (STAI short-form: *M* = 14.6 ± 4.3), psychological inflexibility (AAQ-II: *M* = 19.8 ± 7.2), loneliness (UCLA: *M* = 5.1 ± 1.5), and depressive symptoms (Zung: *M* = 34.7 ± 8.6). The average number of injuries reported in the previous year was 2.3 ± 1.9, with a mean severity of 5.1 ± 2.4 (on a 0–10 scale). Pearson correlation analyses revealed significant associations between neuroticism and depression (*r* = 0.49, *p* < 0.001), anxiety (*r* = 0.61, *p* < 0.001), psychological inflexibility (*r* = 0.38, *p* < 0.01), and loneliness (*r* = 0.27, *p* < 0.05). Injury frequency was significantly correlated with neuroticism (*r* = 0.26, *p* = 0.032) and AAQ-II scores (*r* = 0.23, *p* = 0.041). These relationships are summarized in Table 1.

**Table 1:** Correlation matrix.

Variable	Injury freq.	Injury severity	Neuroticism	STAI	AAQ-II	Zung	UCLA
Injury frequency		0.21*	0.26*	0.18	0.23*	0.16	0.12
Injury severity		1	0.21*	0.15	0.26*	0.34**	0.19
Neuroticism			1	0.61**	0.38**	0.49**	0.27*
STAI				1	0.54**	0.72**	0.25*
AAQ-II					1	0.45**	0.33**
Zung						1	0.29*
UCLA							1

STAI, State-Trait Anxiety Inventory (short-form); AAQ-II, Acceptance and Action Questionnaire-II (psychological inflexibility); Zung, Zung Self-Rating Depression Scale; UCLA, UCLA Loneliness Scale; Injury Freq., number of injuries in the past year; Injury Severity, Self-rated severity of main injury (0–10 scale). *p* < 0.05; *p* < 0.01.

### Predictive analysis: Poisson regression on injury frequency

**Figure 1:**
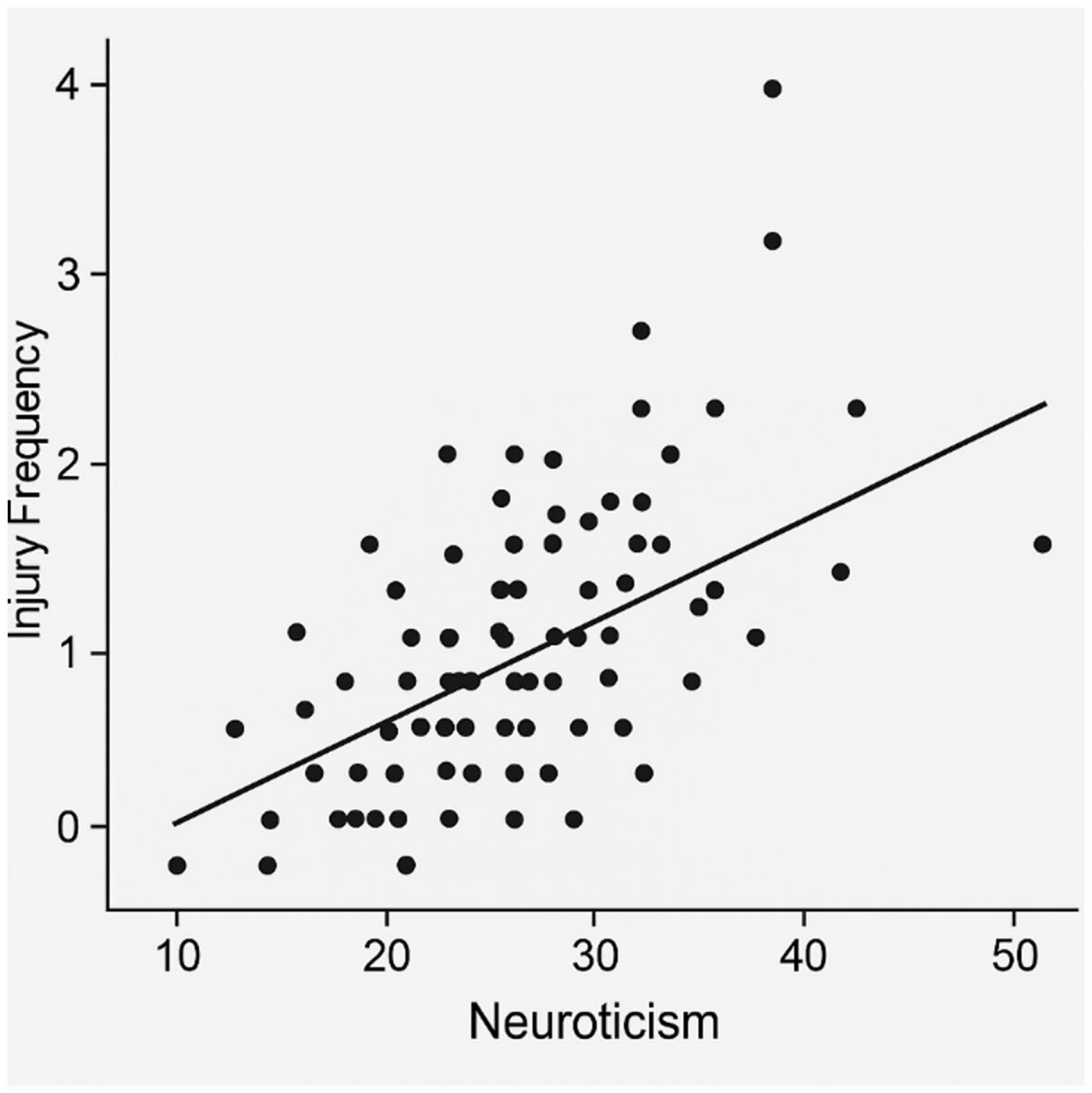
Scatterplot of neuroticism and injury frequency.

A Poisson regression model was conducted with psychological traits as predictors of injury frequency, including neuroticism, AAQ-II, conscientiousness, and STAI, while controlling for age and sex. Neuroticism [Exp (β) = 1.15, *p* = 0.026] and psychological inflexibility [Exp (β) = 1.09, *p* = 0.041] emerged as significant positive predictors. Conscientiousness showed a trend toward a protective effect [Exp (β) = 0.92, *p* = 0.074], while anxiety, age, and sex were not significant. The relationship between neuroticism and injury frequency is visually depicted in Figure 1.

### Linear regression: injury severity

A multiple linear regression analysis was conducted to identify predictors of self-rated injury severity. The model was significant (*F* = 5.3, *p* < 0.001, *R*^2^ = 0.26). Significant predictors included Zung depression scores (β = 0.34, *p* = 0.009), psychological inflexibility (AAQ-II; β = 0.26, *p* = 0.034), and neuroticism (β = 0.21, *p* = 0.048).

### Psychological profiles: cluster analysis

A K-means cluster analysis using neuroticism, conscientiousness, STAI, AAQ-II, UCLA, and Zung scores revealed three distinct psychological profiles:

- Cluster 1 (Resilient): Low neuroticism and anxiety, high conscientiousness; low depression and inflexibility.
- Cluster 2 (Average): Mid-range levels across all traits.
- Cluster 3 (Vulnerable): High neuroticism, anxiety, depression, loneliness, and inflexibility; low conscientiousness.

**Figure 2:**
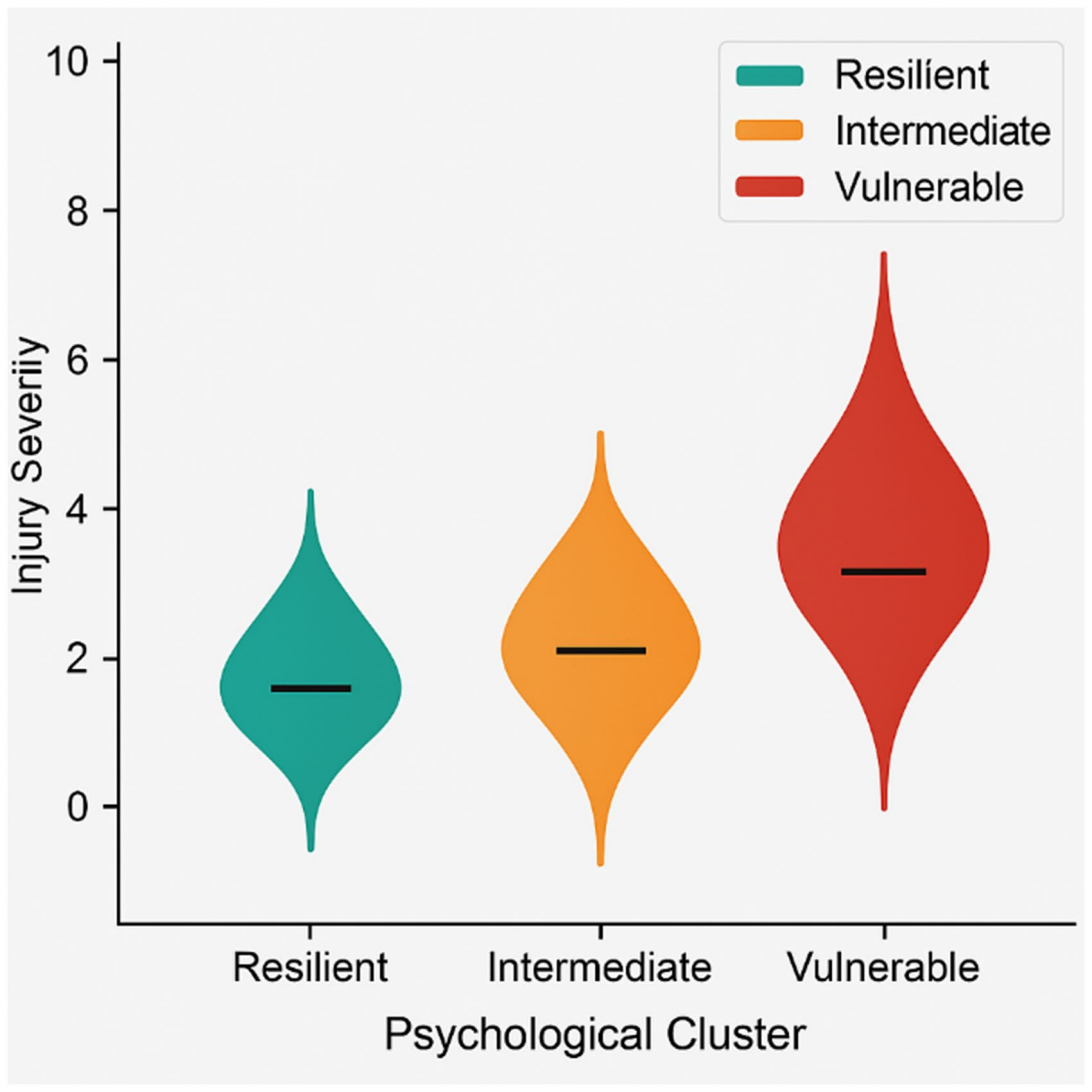
Violin plots of injury severity by psychological cluster.

Group comparisons showed significant differences in injury frequency (*F* = 4.21, *p* = 0.018) and injury severity (*F* = 5.02, *p* = 0.012), with the Vulnerable group exhibiting the highest values. These severity differences are presented in Figure 2.

## Discussion

This study aimed to analyze how psychological variables are related to the frequency and severity of injuries in professional alpine skiers, as well as to explore if certain emotional profiles might be linked to a higher risk of injuries. The results support the idea that specific personality traits and emotional states are significantly associated with both the occurrence and experience of injuries in this sport.

The findings revealed that higher levels of neuroticism and psychological inflexibility were significantly associated with a higher frequency of injuries, while depressive symptoms were linked to higher perceived injury severity. This relationship between emotional vulnerability and sports injuries is consistent with previous studies that have reported similar associations, especially in contexts of high physical and mental demands (Rogers et al., 2024; Stephan et al., 2009).

Psychological inflexibility has been identified as a factor that may limit coping capacity in highly demanding situations. In sports such as alpine skiing, this emotional rigidity might show up as a lack of body awareness, less effective responses to pain or fatigue and an increased chance of encountering risky situations (Johles et al., 2020).

From a theoretical perspective, these findings are consistent with the stress–injury model (Wiese-bjornstal et al., 1998; Andersen and Williams, 1988), which posits that emotional responses and cognitive appraisal in the face of stressors—shaped by characteristics such as neuroticism and coping strategies—can influence attention and decision-making under pressure. Moreover, cognitive-behavioral perspectives suggest that individuals with high levels of neuroticism may exhibit heightened threat sensitivity and a tendency toward rumination, which increases the likelihood of attentional lapses that coincide with the occurrence of injuries (Olmedilla et al., 2018). Likewise, low psychological flexibility reflects a rigid response pattern and greater experiential avoidance, which can hinder the effective management of pain, fatigue, or uncertain situations in alpine skiing (Bond et al., 2011; Ruiz et al., 2013). These mechanisms may help explain why inflexibility and neuroticism often coexist as factors associated with risk. In addition, depressive symptoms may affect the perceived severity of injuries by amplifying negative interpretations of physical sensations and reducing self-efficacy, in line with pain perception and adaptation models (Stephan et al., 2009; Yang et al., 2014).

Regarding the perceived severity of injuries, athletes with higher depression scores also rated their injuries as more severe. This could be explained by a more negative interpretation of physical damage or increased sensitivity to the emotional and social consequences of an injury. This finding aligns with previous studies indicating that changes in emotional states can influence pain perception, adjustment to sport-related rest and rehabilitation adherence (Rogers et al., 2024; Yang et al., 2014; Putukian, 2016).

For the psychological profile analysis, three clearly differentiated groups were identified: one with adaptive traits and low emotional distress (resilient profile), another with intermediate scores (average profile), and a third with high emotional distress and low conscientiousness (vulnerable profile). The latter group was associated with a markedly higher injury burden compared to resilient athletes, suggesting that the combination of certain traits may be critical in understanding injury susceptibility.

The findings of this study become even more significant when we look at recent research on psychophysiological responses to prolonged physical stress during skiing. It has been observed that ski sessions lasting 3 to 4 h are associated with differential effects on fatigue, emotional perception and motor performance (Krautgasser et al., 2012). Moreover, studies in cross-country skiing have pointed out that factors like social isolation, unpredictable weather and high technical demands may negatively influence the development of personality traits and emotional states, such as anxiety or depression (Davey et al., 2019; Broddadóttir et al., 2021). These conditions may act as chronic stressors that, when combined with maladaptive coping structures, contribute to psychological vulnerability and physical injury.

From a practical perspective, these findings highlight the need to integrate psychological assessments into injury prevention programs in high-performance sports. Brief screening tools that evaluate characteristics such as psychological flexibility, anxiety, and neuroticism could be useful for identifying athletes at risk at an early stage. Vulnerability can be reduced by including interventions such as resilience training programs (Stephan et al., 2009), cognitive reappraisal techniques (Birrer and Morgan, 2010), and mindfulness (Yang et al., 2014; Tang et al., 2022), which can enhance emotional regulation and psychological adaptability. Furthermore, these programs could be implemented through brief and specific sessions integrated into daily training routines and coordinated by coaches and sport psychologists (Rogers et al., 2024; Weiß et al., 2024), thereby enabling the development of personalized prevention and rehabilitation strategies.

Nonetheless, some limitations should be acknowledged. The relatively small sample size (*n* = 50), while sufficient according to *a priori* power analysis, may reduce the stability of regression coefficients and limit the robustness of the cluster analysis. Future research with larger samples is needed to replicate and validate these findings. Additionally, the use of retrospective self-report measures for injury outcomes and the operationalization of injury severity through a single self-rated Likert scale introduce potential recall and common-method biases. Moreover, the generalizability of these results may be limited by the cultural and contextual characteristics of the sample (Spanish-speaking elite athletes), and future research should examine whether similar patterns emerge across different competitive levels, countries, and skiing disciplines. Despite these limitations, the present study provides valuable preliminary evidence on the psychological predictors of injury risk and severity in elite alpine skiing and provides a theoretical and empirical foundation for future longitudinal and experimental research in this field.

## Conclusion

The results of this study demonstrate that certain psychological characteristics, such as neuroticism, psychological inflexibility and depressive symptoms, are significantly associated with higher frequency and severity of injuries in professional alpine skiers. Furthermore, the identification of a vulnerable psychological profile provides a deeper understanding of how the interaction of multiple emotional factors can increase injury risk.

These insights highlight the need to weave psychological assessments into injury prevention strategies for elite athletes. Future research should adopt a longitudinal approach, incorporate objective measures of physical strain and recovery and broaden the sample to include athletes from other sports disciplines. The implementation of models that incorporate psychological components may not only enhance injury prevention but also contribute to optimizing athletic performance and athlete wellbeing.

## Data Availability

The original contributions presented in the study are included in the article/supplementary material, further inquiries can be directed to the corresponding author.

## References

[ref1] Andersen M. B. Williams J. M. (1988). A model of stress and athletic injury: prediction and prevention. J. Sport Exerc. Psychol. 10, 294–306. doi: 10.1123/jsep.10.3.294

[ref2] Arbinaga Ibarzábal F. (2025). Pain Catastrophizing related to psychological inflexibility, self-reported injuries and perfectionism in soccer referees. J. Sci. Sport Exerc. 7, 84–96. doi: 10.1007/s42978-023-00234-z

[ref3] Benet-Martínez V. John O. P. (1998). Los Cinco Grandes a través de las culturas: Validez y estructura factorial del BFI en muestras españolas. Rev. Psicol. Gen. Apl.

[ref4] Biggs J. T. Wylie L. T. Ziegler V. E. (1978). Validity of the Zung self-rating depression scale. Br. J. Psychiatry 132, 381–385. doi: 10.1192/bjp.132.4.381, PMID: 638392

[ref5] Birrer D. Morgan G. (2010). Psychological skills training as a way to enhance an athlete’s performance in high-intensity sports. Scand. J. Med. Sci. Sports 20, 78–87. doi: 10.1111/j.1600-0838.2010.01188.x, PMID: 20840565

[ref6] Bond F. W. Hayes S. C. Baer R. A. Carpenter K. M. Guenole N. Orcutt H. K. (2011). Preliminary psychometric properties of the acceptance and action questionnaire–II: a revised measure of psychological inflexibility and experiential avoidance. Behav. Ther. 42, 676–688. doi: 10.1016/j.beth.2011.03.007, PMID: 22035996

[ref7] Broddadóttir E. Flovenz S. Gylfason H. Þormar Þ. Einarsson H. Salkovskis P. (2021). I’m so tired: fatigue as a persistent physical symptom among working people experiencing exhaustion disorder. Int. J. Environ. Res. Public Health 18:8657. doi: 10.3390/ijerph18168657, PMID: 34444405 PMC8392333

[ref8] Burtscher M. Gatterer H. Flatz M. Sommersacher R. Woldrich T. Ruedl G. . (2008). Effects of modern ski equipment on the overall injury rate and the pattern of injury location in alpine skiing. Clin. J. Sport Med. 18, 355–357. doi: 10.1097/MJT.0b013e31815fd0fe, PMID: 18614888

[ref9] Coury T. Napoli A. M. Wilson M. Daniels J. Murray R. (2013). Injury patterns in recreational alpine skiing and snowboarding at a mountainside clinic. Wilderness Environ. Med. 24, 417–421. doi: 10.1016/j.wem.2013.07.002, PMID: 24138836

[ref10] Cuschieri S. (2019). The STROBE guidelines. Saudi J. Anaesth. 13, S31–S34. doi: 10.4103/sja.SJA_543_18 30930717 PMC6398292

[ref11] Davey A. Endres N. K. Johnson R. J. Shealy J. E. (2019). Alpine skiing injuries. Sports Health. 11, 18–26. doi: 10.1177/1941738118813051, PMID: 30782106 PMC6299353

[ref12] Eather N. Wade L. Pankowiak A. E. R. (2023). The impact of sports participation on mental health and social outcomes in adults: a systematic review and the “mental health through sport” conceptual model. Syst. Rev. 12:102. doi: 10.1186/s13643-023-02264-8, PMID: 37344901 PMC10286465

[ref13] Fernández M. E. (1993). Adaptación española de la UCLA Loneliness Scale. Rev. Psicol. Soc.

[ref14] Finkenzeller T. Burberg T. Kranzinger S. Harbour E. Snyder C. Würth S. (2022). Effects of physical stress in alpine skiing on psychological, physiological, and biomechanical parameters: an individual approach. Front. Sports Act. Living. 4:971137. doi: 10.3389/fspor.2022.971137, PMID: 36299402 PMC9589513

[ref15] Gao Y. Che L. Li X. (2024). Running, walking, and cross-country skiing: how to shape adolescents’ personalities through physical activity? Front. Psychol. 15:1489131. doi: 10.3389/fpsyg.2024.1489131 39606205 PMC11600106

[ref16] Guillén-Riquelme A. Buela-Casal G. (2011). Psychometric revision and differential item functioning in the state trait anxiety inventory (STAI). Psicothema 23, 510–515, PMID: 21774907

[ref17] Johles L. Gustafsson H. Jansson-Fröjmark M. Classon C. Hasselqvist J. Lundgren T. (2020). Psychological flexibility among competitive athletes: a psychometric investigation of a new scale. Front. Sport Act Living 2:110. doi: 10.3389/fspor.2020.00110, PMID: 33345099 PMC7739682

[ref18] Junge A. (2000). The influence of psychological factors on sports injuries. Review of the literature. Am. J. Sports Med. 28, S10–S15. doi: 10.1177/28.suppl_5.s-10 11032102

[ref19] Koller A. Fuchs B. Leichtfried V. Schobersberger W. (2015). Decrease in eccentric quadriceps and hamstring strength in recreational alpine skiers after prolonged skiing. BMJ Open Sport Exerc. Med. 1. doi: 10.1136/bmjsem-2015-000028, PMID: 27900115 PMC5117052

[ref20] Korkutata A. Halis M. Bolel B. (2024). The impact of anxiety experienced in competition on decision-making: a study on individual sports competitions. Int. J. Sport. Exerc. Train. Sci. 10, 145–155. doi: 10.18826/useeabd.1532962

[ref21] Krautgasser S. Scheiber P. von Duvillard S. P. Muller E. (2012). Heart rate, mood states, and rating of perceived exertion among elderly subjects during 3.5 hours of recreational alpine skiing. Ann. Kin.

[ref22] Marteau T. M. Bekker H. (1992). The development of a six-item short-form of the state scale of the Spielberger state-trait anxiety inventory (STAI). Br. J. Clin. Psychol. 31, 301–306. doi: 10.1111/j.2044-8260.1992.tb00997.x, PMID: 1393159

[ref23] Martín-Rodríguez A. Tornero-Aguilera J. F. López-Pérez P. J. Clemente-Suárez V. J. (2021). The effect of loneliness in psychological and behavioral profile among high school students in Spain. Sustainability 14:168. doi: 10.3390/su14010168

[ref24] Nicolò A. Massaroni C. Passfield L. (2017). Respiratory frequency during exercise: the neglected physiological measure. Front. Physiol. 8:922. doi: 10.3389/fphys.2017.00922, PMID: 29321742 PMC5732209

[ref25] Olmedilla A. García-Mas A. Ortega E. L. J. S. (2018). Psychological predictors of injury occurrence: a prospective study in professional football. Eur. J. Sport Sci.

[ref26] Olmedilla Zafra A. Garcia-Mas A. (2012). A global psychological model of the sportive injuries. Rev. Psicol. Deporte.

[ref27] Patrick E. Cooper J. G. Daniels J. (2015). Changes in skiing and snowboarding injury epidemiology and attitudes to safety in big sky, Montana, USA: a comparison of 2 cross-sectional studies in 1996 and 2013. Orthop. J. Sports Med. 3:2325967115588280. doi: 10.1177/2325967115588280, PMID: 26665097 PMC4622368

[ref28] Putukian M. (2016). The psychological response to injury in student athletes: a narrative review with a focus on mental health. Br. J. Sports Med. 50, 145–148. doi: 10.1136/bjsports-2015-095586 26719498

[ref29] Rodriguez-Besteiro S. Beltran-Velasco A. I. Tornero-Aguilera J. F. Martínez-González M. B. Navarro-Jiménez E. Yáñez-Sepúlveda R. (2023). Social media, anxiety and COVID-19 lockdown measurement compliance. Int. J. Environ. Res. Public Health 20:4416. doi: 10.3390/ijerph20054416, PMID: 36901425 PMC10001599

[ref30] Rogers D. L. Tanaka M. J. Cosgarea A. J. Ginsburg R. D. Dreher G. M. (2024). How mental health affects injury risk and outcomes in athletes. Sports Health 16, 222–229. doi: 10.1177/19417381231179678, PMID: 37326145 PMC10916780

[ref31] Ruiz F. J. Langer A. I. Luciano C. Cangas A. J. Beltrán I. (2013). Measuring experiential avoidance and psychological inflexibility: the Spanish version of the acceptance and action questionnaire–II. Psicothema 1, 123–129. doi: 10.7334/psicothema2011.239, PMID: 23336554

[ref32] Russell D. W. (1996). UCLA loneliness scale (version 3): reliability, validity, and factor structure. J. Pers. Assess. 66, 20–40. doi: 10.1207/s15327752jpa6601_2 8576833

[ref33] Schneider T. (2003). Snow skiing injuries. Aust. Fam. Physic. 32, 499–502. 12901200

[ref34] Seifert J. Kröll J. Müller E. (2009). The relationship of heart rate and lactate to cumulative muscle fatigue during recreational alpine skiing. J. Strength Cond. Res. 23, 698–704. doi: 10.1519/JSC.0b013e3181a2b55e, PMID: 19387412

[ref35] Stenroos A. Handolin L. (2015). Incidence of recreational alpine skiing and snowboarding injuries: six years experience in the largest ski resort in Finland. Scand. J. Surg. 104, 127–131. doi: 10.1177/1457496914532249, PMID: 24786173

[ref36] Stephan Y. Deroche T. Brewer B. Caudroit J. Scanff C. L. (2009). Predictors of perceived susceptibility to sport-related injury among competitive runners: the role of previous experience, neuroticism, and passion for running. Appl. Psychol. 58, 672–687. doi: 10.1111/j.1464-0597.2008.00373.x

[ref37] Tang Y. Liu Y. Jing L. Wang H. Yang J. (2022). Mindfulness and regulatory emotional self-efficacy of injured athletes returning to sports: the mediating role of competitive state anxiety and athlete burnout. Int. J. Environ. Res. Public Health 19:11702. doi: 10.3390/ijerph191811702, PMID: 36141969 PMC9517234

[ref38] Weiß M. Büttner M. Richlan F. (2024). The role of sport psychology in injury prevention and rehabilitation in junior athletes. Behav. Sci. 14:254. doi: 10.3390/bs14030254, PMID: 38540557 PMC10968622

[ref39] Wiese-bjornstal D. M. Smith A. M. Shaffer S. M. Morrey M. A. (1998). An integrated model of response to sport injury: psychological and sociological dynamics. J. Appl. Sport Psychol. 10, 46–69. doi: 10.1080/10413209808406377

[ref40] Xiao D. Z. Q. (2024). Exploring performance of athletic individuals: tying athletic behaviors and big-five personality traits with sports performance. PLoS One 19:e0312850. doi: 10.1371/journal.pone.0312850, PMID: 39621699 PMC11611207

[ref41] Yang J. Cheng G. Zhang Y. Covassin T. Heiden E. O. Peek-Asa C. (2014). Influence of symptoms of depression and anxiety on injury hazard among collegiate American football players. Res. Sport. Med. 22, 147–160. doi: 10.1080/15438627.2014.881818, PMID: 24650335

[ref42] Zung W. W. (1965). Self-rating depression scale. Arch. Gen. Psychiatry 22, 63–70. doi: 10.1001/archpsyc.1965.01720310065008 14221692

