# Correction: Psychological predictors of sports injuries in elite ski athletes: a multidimensional analysis of personality, anxiety, depression and inflexibility

**DOI:** 10.3389/fpsyg.2025.1740151

**Published:** 2025-11-20

**Authors:** Sara Mogedano-Cruz, Vicente Javier Clemente-Suárez, Rafael Jácome-López, Fernando García-Sanz, Laura González-Fernández, Angel González-de-la-Flor, Carlos Romero-Morales

**Affiliations:** Universidad Europea de Madrid, Faculty of Medicine, Health and Sports, Department of Physiotherapy, Villaviciosa de Odón, Madrid, Spain

**Keywords:** alpine skiing, sports injuries, neuroticism, mental health, psychological profile, injury prevention

The title of this article was erroneously given as: “Psychological predictors of sports injuries in elite sky athletes: a multidimensional analysis of personality, anxiety, depression and inflexibility.” The correct title of the article is “Psychological predictors of sports injuries in elite ski athletes: a multidimensional analysis of personality, anxiety, depression and inflexibility.”

The original version of this article has been updated.

